# WHO antibody reference panel for donor screening assays that detect antibodies to human T-lymphotropic virus type 1 (HTLV-1) and type 2 (HTLV-2)

**DOI:** 10.1186/1742-4690-8-S1-A246

**Published:** 2011-06-06

**Authors:** Erin Wigglesworth, Elliot P Cowan, Clare Morris

**Affiliations:** 1 Division of Retrovirology, NIBSC, Potters Bar, Hertfordshire, EN6 3QG, UK; 2 Division of Emerging and Transfusion Transmitted Diseases, Center for Biologics Evaluation and Research, US FDA, Rockville, Maryland, 20852, USA

## 

Antibody screening tests are critical tools to identify individuals infected with HTLV-1 and HTLV-2. However, the lack of reference panels hinders the ability to evaluate existing tests and new technologies that aid in the screening for these agents. For example, some HTLV-2 subtypes may escape detection by currently available technology. The World Health Organization, through the support of the WHO Expert Committee on Biological Standardization, is in the process of generating such a reference panel.

This reference panel will best function as a tool for test assessment only if it consists of specimens representing the diversity of HTLV-1 and HTLV-2, especially those subtypes known to present challenges to detection using serological tests. A panel consisting of seven members (HTLV-1a-d and HTLV-2a,b,d) is being considered.

The reference panel will be fully characterised and assessed in an international collaborative study. This first WHO HTLV antibody reference panel is expected to be used by blood screening and diagnostic test developers, blood banks, hospitals and other establishments performing HTLV serology testing.

